# Data on the fate of MACS® MicroBeads intramyocardially co-injected with stem cell products

**DOI:** 10.1016/j.dib.2017.06.035

**Published:** 2017-06-24

**Authors:** Paula Müller, Ralf Gaebel, Heiko Lemcke, Gustav Steinhoff, Robert David

**Affiliations:** a Reference and Translation Center for Cardiac Stem Cell Therapy (RTC), Department of Cardiac Surgery, Rostock University Medical Center, Schillingallee 69, 18057 Rostock, Germany; b Department Life, Light and Matter of the Interdisciplinary Faculty at Rostock University, Albert-Einstein Straße 25, 18059 Rostock, Germany

**Keywords:** Stem cell therapy, Cardiovascular regeneration, Haematopoietic stem cells (HSCs), Mesenchymal stem cells (MSCs), Magnetic activated cell sorting (MACS®), MACS® MicroBeads

## Abstract

The data presented in this article are related to the research article “Intramyocardial Fate and Effect of Iron Nanoparticles co-injected with MACS® purified Stem Cell Products” (Müller et al., 2017) [1]. This article complements the cellular localization of superparamagnetic iron dextran particles (MACS® MicroBeads) used for magnetic activated cell sorting (MACS®). Data evaluate the time-dependent detachment of these nanoparticles from CD133^+^ haematopoietic stem cells (HSCs) and CD271^+^ mesenchymal stem cells (MSCs). Furthermore, the influence of these stem cells as well as of nanoparticles on cardiac remodeling processes after myocardial infarction (MI) was investigated.

## **Specifications Table**

**Table:** 

Subject area	*Biology*
More specific subject area	*Intramyocardial transplantation of MACS® purified stem cell products*
Type of data	*Image, graph, figure, text file*
How data was acquired	*Structured illumination microscopy (Zeiss ELYRA PS.1 LSM 780), flow cytometry (BD LSR-II), histological staining*
Data format	*Analyzed*
Experimental factors	*CD133*^*+*^*and CD271*^*+*^*stem cells were automatically (using the CliniMACS® Prodigy BM-133 system) and manually (using Mini MACS® technology) isolated from human bone marrow (BM)*
Experimental features	*Investigation of the Intracellular localization and time-dependent detachment of MACS® MicroBeads from stem cells using the Labeling Check Reagent-FITC (Miltenyi Biotec). Impact of MACS® MicroBeads on collagen deposition after myocardial infarction using an ischemia/reperfusion mouse model and Sirius Red staining.*
Data source location	*Rostock University Medical Center, Schillingallee 69, 18057 Rostock, Germany*
Data accessibility	*The data are available with this article*

## **Value of the data**

• MACS® is the most commonly used technique for the purification of stem cell subpopulations intended for the treatment of cardiovascular diseases.

• Data about the binding of MACS® MicroBeads to stem cells are crucial for *in vivo* application of stem cell products.
• Data provide information about the effect of co-injected MACS® MicroBeads on cardiac remodeling processes after MI.
• Data can be useful for other researchers analyzing the cardiac regeneration potential of MACS® purified stem cells products.
• Data clarifies the safety of MACS® MicroBeads for clinical application.

## 1 Data

**Fig. 1:**
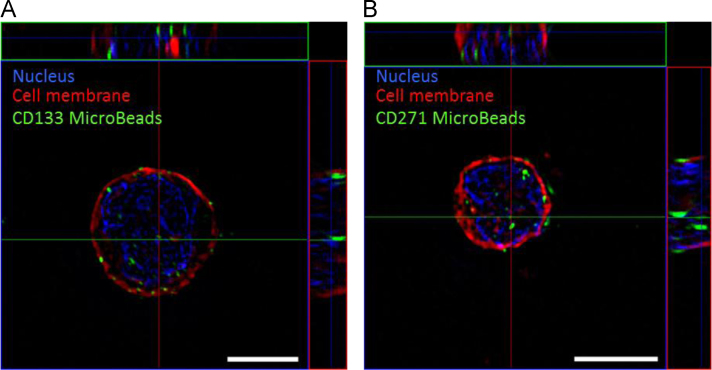
Cellular localization of MACS® MicroBeads in stem cells. CD133^+^**(A)** and CD271^+^**(B)** stem cells were isolated from human bone marrow using manual MACS® technology. Staining of CD133 and CD271 MACS® MicroBeads was performed with Labeling Check Reagent-FITC (green). Cell membrane was stained with CellMask™ Plasma Membrane Stains (red). Nuclei were counterstained with DAPI (blue). Representative z-stack acquisition was performed using structured illumination microscopy (SIM). Scale bar = 5 µm.

**Fig. 2:**
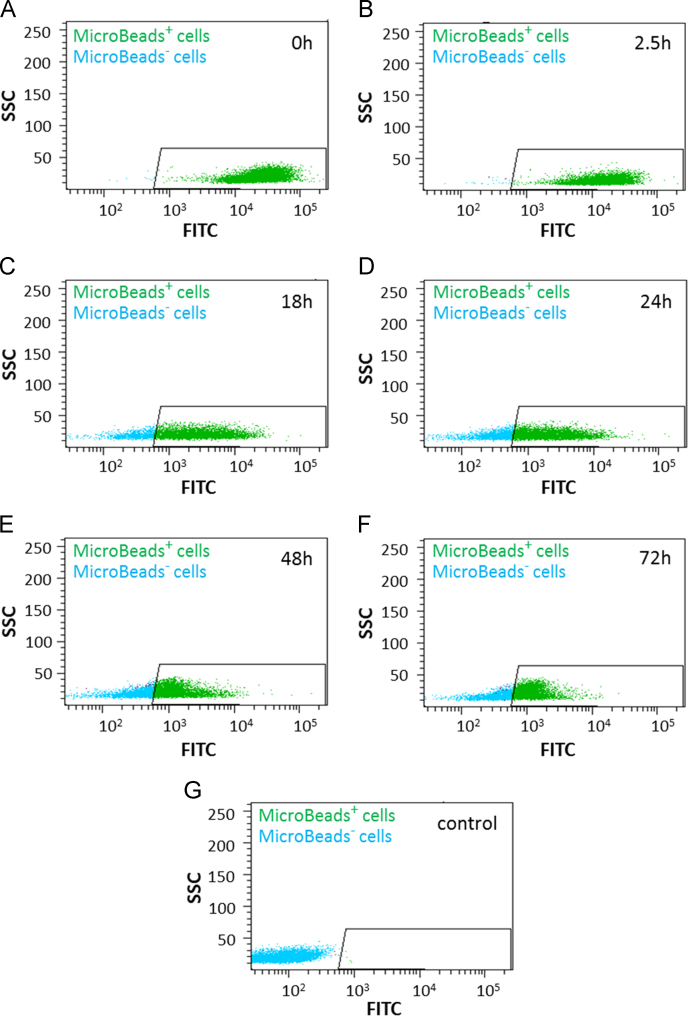
Representative flow cytomtry plots showing the fluorescence intensity of MACS® MicroBeads-FITC labelled cells. CD133^+^ cells were isolated using manual MACS® technology and incubated under cell culture conditions at 37 °C. At respective time points (0 h **(A)**; 2.5 h **(B)**; 18 h **(C)**; 24 h **(D)**; 48 h **(E)**; 72 h **(F)**) MACS® MicroBead staining was performed with Labeling Check Reagent-FITC and fluorescence intensity was measured by flow cytometry. Unstained cells were used as control (G). Green: Cells positive for MACS® MicroBeads. Blue: Cells negative for MACS® MicroBeads.

**Fig. 3:**
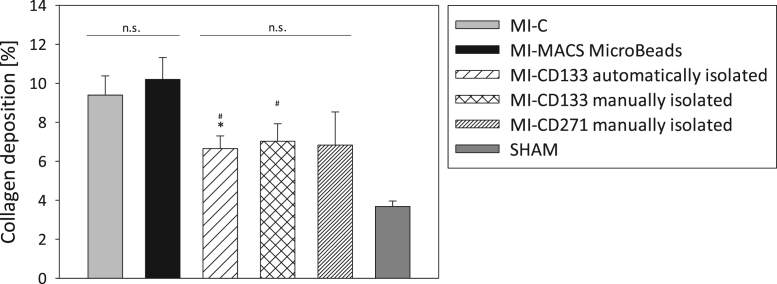
Collagen deposition after intramyocardial transplantation of MACS® MicroBeads and stem cells. MACS® MicroBeads or 1×10^5^ CD133^+^ and CD271^+^ stem cells were intramyocardially injected into SCID *bg* mice after myocardial infarction (MI). Three weeks after transplantation, fibrotic events at the infarction border zone were analyzed by histological staining of heart slices with Sirius Red and Fast Green FCF. For control, untreated infarction (MI-C) and SHAM operation were used. Collagen deposition is expressed as the ratio of collagen deposition to myocardial tissue in percentage. Values are presented as mean±SEM; *n* = 7 (MI-C; MI-CD133 manually isolated; MI-CD271 manually isolated); *n* = 6 (MI-MACS MicroBeads; MI-CD133 automatically isolated; SHAM); ∗ and # = *p* ≤ 0.05; ∗∗ and ## = *p* ≤ 0.01; ∗∗∗ and ### = *p* ≤ 0.001 vs. MI-C (∗) or MI-MACS MicroBeads (#).

The data include information about the cellular localization of MACS® MicroBeads (labelled with Labeling Check Reagent-FITC) right after the manual MACS® based isolation of CD133^+^ and CD271^+^ stem cells (Fig. 1). The detachment of FITC-labelled MACS® MicroBeads was evaluated by measuring the time-dependent fluorescence intensity of MACS® purified CD133^+^ cells incubated under cell culture conditions (37 °C in StemSpan^TM^ H3000) using flow cytometry (Fig. 2). Furthermore, the effect of manually and automatically (Good Manufacturing Practice (GMP)-conform) MACS® purified CD133^+^ and CD271^+^ stem cells as well as of MACS® MicroBeads on fibrosis after MI was assessed in a cardiac ischemia/reperfusion mouse model by histological staining (Fig. 3).

## 2 Experimental design, materials and methods

### 2.1 Sternal BM harvesting

Sternal BM aspirates were obtained as previously described [2].

### 2.2 CD133^+^ and CD271^+^ cell isolation

CD133^+^ and CD271^+^ cells were automatically and manually isolated as previously described [1], [3].

### 2.3 Microscopic analysis

For staining, cells were incubated with Labeling Check Reagent-FITC (Miltenyi Biotec) and CellMask^TM^ Plasma Membrane Stains (Thermo Fisher Scientific, Schwerte, Germany). Subsequently, samples were mounted with Fluoroshield™ with DAPI (Sigma-Aldrich, Taufkirchen, Germany) on microscope slides. To evaluate the localization of MACS® MicroBeads, labelled cells were subjected to three-dimensional structured illumination microscopy (SIM) using the ELYRA PS.1 LSM 780 system (Carl Zeiss, Jena, Germany). Images were acquired as z-stacks and processed with ZEN software (Carl Zeiss). Final images were obtained by creation of maximum projections.

### 2.4 Assay to address the detachment of MACS® MicroBeads

Mean fluorescence of MACS® MicroBeads labelled CD133^+^ cells was measured using flow cytometry. At respective time points samples were taken from cultured cells and incubated with human FcR Blocking Reagent (Miltenyi Biotec), CD133/2 (293C3)-PE antibody (Miltenyi Biotec), 7-Amino-Actinomycin (7-AAD) staining solution (Becton Dickinson, Heidelberg, Germany) and Labeling Check Reagent-FITC. Samples were measured using BD LSR-II flow cytometer (Becton Dickinson) and row data were analysed with FACSDiva software version 6.1.2 (Becton Dickinson).

### 2.5 Generation of cardiac ischemia/reperfusion and intramyocardial injections

This study was approved by the federal animal care committee of the Landesamt für Landwirtschaft, Lebensmittelsicherheit und Fischerei Mecklenburg-Vorpommern (LALLF, Germany) (LALLF M-V/TSD/7221.3-1.1-088/11). To simulate MI, severe combined immunodeficiency beige (SCID bg) mice (CB17.Cg-Prkdc^scid^Lyst^bg-J^/Crl) were anesthetized and after thoracotomy the left anterior descending coronary artery (LAD) was ligated. After 45 min, each mouse received an intramyocardial application of 1×10^5^ cells or 1.3 µl of CD133 MACS® MicroBeads mixed with Growth Factor Reduced (GFR) Matrigel^TM^ Matrix (Corning, Berlin, Germany). The untreated MI control group (MI-C) underwent the same surgical treatment with GFR Matrigel^TM^ Matrix application. Injections were performed along the border of the blanched myocardium and LAD ligation was removed. The healthy control group (SHAM) underwent identical surgical procedures as the MI-C group without LAD ligation.

### 2.6 Histological investigations

Three weeks after cardiac surgery, murine hearts were embedded in Tissue-Tek® O.C.T.^TM^ Compound (Zoeterwoude, Netherlands) and snap-frozen. To investigate fibrosis, 5 µm thick slices were cut from two different horizontal myocardial infarction levels and stained with Sirius Red (Division Chroma, Muenster, Germany) and Fast Green FCF (Sigma-Aldrich). Sirius Red positive regions (indicating collagen deposition) were examined in the infarction border zone (BZ) in five randomly chosen fields (each per section; one section per level; 400×) using computerized planimetry.

### 2.7 Statistical analysis

All statistical analyses were performed using SigmaPlot 11.0 (Systat Software GmbH, Germany). Student׳s t-test was applied to determine the significance. All values are presented as mean ± standard error of the mean (SEM).

## Funding source

This work was supported by the Federal Ministry of Education and Research Germany (FKZ 0312138A and FKZ 316159), the State Mecklenburg-Western Pomerania with EU Structural Funds (ESF/IVWM-B34-0030/10 and ESF/IVBM-B35-0010/12), and the DFG (DA1296-1) and the German Heart Foundation (F/01/12). In addition, R.D. is supported by the FORUN Program of Rostock University Medical Centre (889001), the DAMP Foundation (2016-11)and the BMBF (VIP + 00240).

## References

[1] Müller P., Gaebel R., Lemcke H., Wiekhorst F., Hausburg F., Lang C., Zarniko N., Westphal B., Steinhoff G., David R. (2017). Intramyocardial fate and effect of iron nanoparticles co-injected with MACS® purified stem cell products. Biomaterials.

[2] Müller P., Voronina N., Hausburg F., Lux C.A., Wiekhorst F., Steinhoff G., David R. (2016). Magnet-bead based microRNA delivery system to modify CD133+ stem cells. Stem Cells Int..

[3] Skorska A., Müller P., Gaebel R., Grosse J., Lemcke H., Lux C.A., Bastian M., Hausburg F., Zarniko N., Bubritzki S., Ruch U., Tiedemann G., David R., Steinhoff G. (2017). GMP-conformant on-site manufacturing of a CD133+ stem cell product for cardiovascular regeneration. Stem cell Res. Ther..

